# Racial bias in clinician assessment of patient credibility: Evidence from electronic health records

**DOI:** 10.1371/journal.pone.0328134

**Published:** 2025-08-13

**Authors:** Mary Catherine Beach, Keith Harrigian, Brant Chee, Alya Ahmad, Anne R. Links, Ayah Zirikly, Dingfen Han, Emily Boss, Shari Lawson, Mustapha Saheed, Yahan Li, Mark Dredze, Somnath Saha

**Affiliations:** 1 Department of Medicine, School of Medicine, Johns Hopkins University, Baltimore, Maryland, United States of America; 2 Center for Health Equity, Johns Hopkins University, Baltimore, Maryland, United States of America; 3 Berman Institute of Bioethics, Johns Hopkins University, Baltimore, Maryland, United States of America; 4 Department of Computer Science, Whiting School of Engineering, Johns Hopkins University, Baltimore, Maryland, United States of America; 5 Applied Physics Lab, Johns Hopkins University, Baltimore, Maryland, United States of America; 6 Department of Otolaryngology, School of Medicine, Johns Hopkins University, Baltimore, Maryland, United States of America; 7 Department of Obstetrics and Gynecology, School of Medicine, Howard University, Washington, District of Columbia, United States of America; 8 Department of Emergency Medicine, School of Medicine, Johns Hopkins University, Baltimore, Maryland, United States of America; University of Missouri School of Medicine, UNITED STATES OF AMERICA

## Abstract

**Objective:**

Black patients disproportionately report feeling disbelieved or having concerns dismissed in medical encounters, suggesting potential racial bias in clinicians’ assessment of patient credibility. Because this bias may be evident in the language used by clinicians when writing notes about patients, we sought to assess racial differences in use of language either undermining or supporting patient credibility within the electronic health record (EHR).

**Methods:**

We analyzed 13,065,081 notes written between 2016–2023 about 1,537,587 patients by 12,027 clinicians at a large health system with 5 hospitals and an extensive network of ambulatory practices in the mid-Atlantic region of the United States. We developed and applied natural language processing models to identify whether or not a note contained terms undermining or supporting patient credibility, and used logistic regression with generalized estimating equations to estimate the association of credibility language with patient race/ethnicity.

**Results:**

The mean patient age was 43.3 years and 55.9% were female; 57.6% were non-Hispanic White, 28.0% non-Hispanic Black, 8.3% Hispanic/Latino, and 6.1% Asian. Clinician-authors were attending physicians (44.9%), physicians-in-training (40.1%) and advanced practice providers (15.0%). Terms specifically related to patient credibility were relatively uncommon, with 106,523 (0.82%) notes containing terms undermining patient credibility, and 33,706 (0.26%) supporting credibility. In adjusted analyses, notes written about non-Hispanic Black vs. White patients had higher odds of containing terms undermining credibility (aOR 1.29, 95% CI 1.27–1.32), and lower odds of supporting credibility (aOR 0.82; 95% CI 0.79–0.85). Notes written about Hispanic/Latino vs. White patients had similar odds of language undermining (aOR 0.99, 95% CI 0.95–1.03) and supporting credibility (aOR 0.95, 95% CI 0.89–1.02). Notes written about Asian vs. White patients had lower odds of language undermining credibility (aOR 0.85, 95% CI 0.81–0.89), and higher odds of supporting credibility (aOR 1.30, 95% CI 1.23–1.38).

**Conclusions:**

Clinician documentation undermining patient credibility may disproportionately stigmatize Black individuals and favor Asian individuals. As stigmatizing language in medical records has been shown to negatively influence clinician attitudes and decision making, these racial differences in documentation may influence patient care and outcomes and exacerbate health inequities.

## Introduction

There is mounting evidence that electronic health records (EHR) contain language reflecting the unconscious biases of clinicians, [1–7] and that this language may undermine the quality of care that patients receive.[8,9] Most of these studies have focused on stigmatizing language broadly, characterizing numerous ways that patients are negatively portrayed [1,2,10] and documenting racial disparities in the prevalence of negative or stigmatizing language [3–7].

One category of negative language that receives limited attention relates to assessments of patient credibility. Credibility assessments reflect a very specific type of bias, termed testimonial injustice, wherein a person (e.g., patient) may be less likely to be believed due to a prejudice on the part of the listener (e.g., clinician) [11]. Several qualitative studies suggest that Black Americans are more likely to have their concerns dismissed in healthcare settings [12,13], and two recent studies have documented this phenomenon by examining medical records. In our own earlier work [14], we found that notes from an academic ambulatory internal medicine clinic for patients identified as Black compared to White contained more markers of doubt in each of three linguistic categories: evidentials reporting the patient’s history as hearsay, patient quotes, and specific ‘doubt’ words. A recent study by Lee and colleagues [15] in an inpatient internal medicine setting similarly found greater use of evidentials in the notes of Black compared to White patients. While these early studies provide suggestive evidence of testimonial injustice in the EHR, they have been limited to the field of internal medicine and have used methods with limited specificity in identifying credibility-related language.

In the current study we sought to expand and strengthen the evidence related to bias in credibility assessments documented in the EHR in three important ways. First, we focused specifically on words used to undermine patient credibility using highly reliable natural language processing (NLP) models. Second, using these NLP models, we further distinguished different categories of terms undermining credibility, including those that cast doubt on patients’ sincerity as well as terms that undermine or support patients’ competence in providing accurate information (poor/good historian). Finally, we expanded beyond the field of internal medicine to include a wider range of clinical disciplines. Our overall goal was to assess possible racial differences in clinician use of language either supporting or undermining patient credibility within the EHR of patients from a large health system.

## Methods

### Study design, subjects, and setting

We conducted a cross-sectional analysis of notes written between 1/1/2016 and 10/30/2023 in four clinical fields – emergency medicine, internal medicine, obstetrics & gynecology (OB/GYN), and surgery – including subspecialties within each discipline – at a large academic health system, including a diverse group of 5 hospitals and hospital-based ambulatory practices as well as a network of 15 community-based practices across the mid-Atlantic region of the United States. Notes were included if they were: 1) written about a patient >=18 years of age identified in the EHR as non-Hispanic Black, Hispanic/Latino, non-Hispanic Asian, or non-Hispanic White; 2) written by an attending physician, a physician-in-training (medical students, residents, fellows), or an advanced practice provider (APP, including nurse practitioners and physician assistants) in one of the four clinical departments across the health system; and 3) in a note type containing free (vs. only templated) text (e.g., History & Physicals, Progress Notes, Discharge Summaries, and Consults). The study was reviewed and approved with a waiver of informed consent by the Johns Hopkins Institutional Review Board (IRB00363939). Because this retrospective review of medical records contained protected health information, all data were stored on an approved, secure, and compliant platform; only study team members had access. The data for this analysis were first accessed on February 14, 2024.

### Identification of credibility language

We developed a conceptual framework for credibility language based on previous analyses of medical records [1,14] informed by the theory of epistemic injustice [11], wherein assessments of credibility are made based on either competence (whether or not the person is capable of relaying valuable information) or sincerity (whether or not a person is likely to be lying). We conceptualized assessments of patient competence to be made based on documentation of how the patient is characterized as a *historian*, with modifiers such as poor, unreliable, challenging, limited, inconsistent, etc. undermining competence, and modifiers such as excellent, very good, good, reliable, detailed, etc. supporting patient competence. Based on earlier work [14], language undermining credibility on the basis of sincerity included all forms of the word *claims* (e.g., “claiming that Tylenol does not work for her”), *insists* (e.g., “she insisted she took her medication”), *adamant* (e.g., “he adamantly denied smoking”), as well as any documentation of *drug* or *narcotic seeking*, *malingering*, or *secondary gain*. Although our previous work included two additional methods of casting doubt on patients’ testimonies (use of quotes and evidentials), we did not include those in this analysis because those linguistic features have lower specificity, meaning that they are often used in ways that do not explicitly express doubt.

### Development of natural language processing models

We developed natural language processing (NLP) models to detect credibility language within clinical notes. Our NLP development process is described in detail elsewhere [16]. Briefly, we extracted over 10,000 excerpts from clinical notes from 2 different health systems that included stigmatizing terms, 2,337 of which included one or more of the credibility-related terms described above. The excerpts were reviewed by at least 2 reviewers and coded for inclusion or exclusion. For instance, “he insists he has stopped drinking” would be coded as related to credibility, whereas “he insists on being seen by a cardiologist” would be coded as unrelated, because it casts the patient as demanding rather than lacking credibility. Likewise, excerpts including “not a good historian” were coded as undermining credibility despite using the words “good historian.” Prior to full-scale annotation, we used a small sample of notes to resolve discrepancies and develop an annotation guide for each term in our dictionary. Inter-annotator agreement was high (Krippendorff’s α 0.87), and disagreements were resolved by consensus with a 3^rd^ reviewer [16]. We then used this annotated dataset, employing a 70/20/10% split of the excerpts to train, refine, and validate, respectively, a machine learning classifier to correctly identify credibility-related statements. We used a Clinical BERT NLP model, [17] which had 92% overall accuracy and macro-F1 scores from 0.70 to 0.89.[16] Descriptions and code for our NLP models can be accessed at https://github.com/kharrigian/ehr-stigma.

### Statistical analysis

We created a dichotomous variable indicating whether there was one or more instances of language within each category in each note. We used descriptive analyses to characterize our study sample, and then t-tests and χ^2^ tests to examine the association of patient race/ethnicity with other patient demographic characteristics (age, gender, insurance, interpreter use) and variables related to clinical context (diagnosis of substance use disorder [SUD] [18] or severe mental illness [SMI] [19,20], clinical department, and clinician role/training level). We adjusted for SUD and SMI to account for potential confounding by these conditions, as both were associated with patient race and with use of terms questioning credibility. ICD codes defining SUD and SMI are included in the S1 Appendix. We conducted logistic regression analyses to estimate the odds of each type of language based on patient race/ethnicity, using generalized estimating equations to account for clustering of notes within patient-clinician dyads. We used patient-clinician dyads to account for clustering of notes within patients and clinicians due to the non-hierarchical nature of our data; notes for a given patient could have been written by multiple clinicians, making hierarchical analytic methods (notes within patients and patients within clinicians) inappropriate. We first executed unadjusted models, then added patient demographic characteristics to the model, and finally added clinical context variables. Data were complete for all variables except insurance (0.5% missing) and interpreter use (0.002% missing); multivariate analyses excluded these few notes with missing data. Finally, after examining patterns uncovered by the primary analysis, we conducted separate exploratory models examining the independent association of each word group used to undermine credibility (adamant, claims, insists, malingering, narcotic/drug seeking, secondary gain, poor historian), comparing the notes of Black vs. non-Black patients. All analyses were conducted using Stata (Version 18.0, StataCorp. 2023. College Station, TX, 77845 USA).

## Results

### Study sample

There were 13,065,081 notes written about 1,537,587 patients by 12,027 clinicians. Clinician-authors were attending physicians (44.9%), physicians in training (40.1%) and advanced practice providers (15.0%). Characteristics of patients and notes are shown in Table 1. The mean patient age was 43.3 years and 56% of patients were female. More than half (57.6%) were non-Hispanic White, 28.0% were non-Hispanic Black, 8.3% Hispanic/Latino, and 6.1% Asian. Most patients had commercial insurance (58.7%); 19.2% had Medicare, 14.9% had Medicaid and 7.2% were uninsured. Most notes were written for encounters in the Internal Medicine or Surgery departments (46.4 and 30.1% respectively), and most were written by attending physicians (65.7%). There were significant differences by patient race/ethnicity for all demographic and clinical variables, as outlined in Table 1.

**Table 1:** Participant characteristics.

	Patients	Notes	Notes by Patient Race/Ethnicity^			
	n = 1,537,587	n = 13,065,081	White n = 7,439,604	Black n = 4,162,957	Hispanic/ Latino n = 814,542	Asian n = 647,978
Age*, mean (SD)	43.3(23.2)	52.9(21.1)	56.5(20.5)	49.5(20.3)	39.7(20.6)	49.6(21.2)
**Gender, n (%)**						
Male	677182(44.0)	5502777(42.1)	3307238(44.5)	1642931(39.5)	313640(38.5)	238968(36.9)
Female	859772(55.9)	7559870(57.9)	4130966(55.5)	2519457(60.5)	500540(61.5)	408907(63.1)
Nonbinary	182(0.01)	1241(0)	943(0)	159(0)	69(0)	70(0)
Other/unknown	451(0.01)	1193(0)	457(0)	410(0)	293(0)	33(0)
**Race/Ethnicity, n (%)**						
White	885590(57.6)	7439604(56.9)				
Black	430921(28)	4162957(31.9)				
Hispanic/Latino	126803(8.3)	814542(6.2)				
Asian	94273(6.1)	647978(5.0)				
**Interpreter, n (%)**						
No	1470736(95.7)	12567417(96.2)	7392946(99.4)	4137561(99.4)	479268(58.9)	557642(86.1)
Yes	66828(4.4)	497417(3.8)	46634(0.6)	25387(0.6)	335063(41.2)	90333(13.9)
**Insurance, n (%)**						
Commercial	901868(58.7)	6335794(48.7)	3862496(52.3)	1709425(41.2)	352795(43.4)	411078(63.6)
Medicare	294324(19.2)	4277714(32.9)	2829855(38.3)	1194611(28.8)	97996(12.1)	155252(24)
Medicaid	228891(14.9)	1873318(14.4)	534611(7.2)	1088579(26.2)	195710(24.1)	54418(8.4)
Uninsured	110462(7.2)	515290(4.0)	163688(2.2)	159770(3.9)	166448(20.5)	25384(3.9)
**Comorbidities**						
Substance Use Disorder, n (%)	68561(4.5)	1459906(11.2)	691373(9.3)	690860(16.6)	61644(7.6)	16029(2.5)
Severe Mental Illness, n (%)	35917(2.3)	709791(5.4)	358421(4.8)	319407(7.7)	21262(2.6)	10701(1.7)
**Department, n (%)**						
Emergency	539025(35.1)	1694525(13)	707250(9.5)	733325(17.6)	183506(22.5)	70444(10.9)
Internal Medicine	418856(27.2)	6056327(46.4)	3569439(48.0)	1954379(47.0)	267016(32.8)	265493(41.0)
OB/GYN	118510(7.7)	1378000(10.6)	655346(8.8)	445060(10.7)	156493(19.2)	121101(18.7)
Surgery	461196(30.0)	3936229(30.1)	2507569(33.7)	1030193(24.8)	207527(25.5)	190940(29.5)
**Clinician, n (%)**						
Attending	5398(44.9)	8587461(65.7)	5048379(67.9)	2548457(61.2)	508470(62.4)	482155(74.4)
Trainee	4823(40.1)	2137607(16.4)	1042170(14)	875191(21)	150240(18.4)	70006(10.8)
APP	1806(15.0)	2340013(17.9)	1349055(18.1)	739309(17.8)	155832(19.1)	95817(14.8)

^All p-values <0.001; * age in years

### Language undermining patient credibility

Fewer than 1% of notes contained language undermining patient credibility (n = 106,523; 0.82%), with 62,480 (0.48%) containing language undermining patients’ sincerity and 52,243 (0.40%) containing language undermining competence.

**Fig 1:**
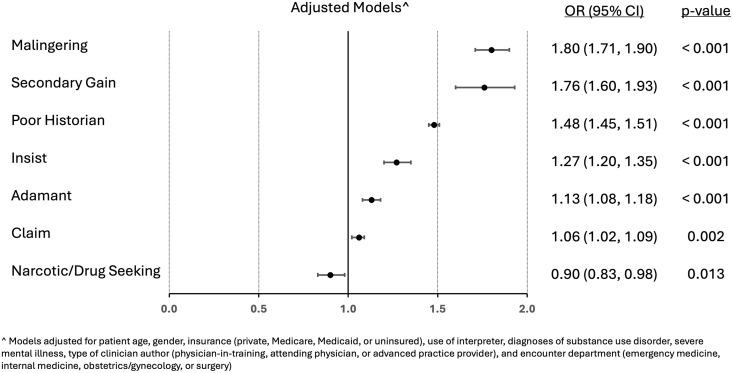
Differences in specific terms undermining credibility for black vs. non-black patients.

In adjusted analyses (Table 2), notes written about non-Hispanic Black vs. White patients had overall higher odds of containing terms undermining credibility (aOR 1.29, 95% CI 1.27–1.32). This disparity was observed for terms undermining both patients’ sincerity (aOR 1.16; 95% CI 1.14–1.19) and patients’ competence (aOR 1.50; 95% 1.47–1.54). In terms of differences in specific words used in notes of Black vs. non-Black patients (Fig 1), there was a higher odds of each individual word undermining patient credibility, except for the terms related to narcotic or drug-seeking, which had a lower odds of appearing in the notes of Black vs. non-Black patients.

**Table 2:** Racial/ethnic differences in language undermining and supporting credibility^1^ in 13,681,829 notes across 4 clinical departments.

			n	%	Unadjusted^1^			Adjusted Model 1^1,2^			Adjusted Model 2^1,3^		
					OR	95% CI		OR	95% CI		OR	95% CI	
Undermining Credibility	ANY	White	53,616	0.76	1.00	*Reference*		1.00	*Reference*		1.00	*Reference*	
		Black	45,536	1.18	1.55	1.53	1.57	1.46	1.43	1.48	1.29	1.27	1.32
		Hispanic	4,570	0.65	0.84	0.81	0.87	0.93	0.89	0.97	0.99	0.95	1.03
		Asian	2,801	0.46	0.63	0.60	0.66	0.73	0.70	0.77	0.85	0.81	0.89
	Sincerity	White	31,167	0.42	1.00	*Reference*		1.00	*Reference*		1.00	*Reference*	
		Black	27,223	0.65	1.65	1.61	1.68	1.31	1.28	1.34	1.16	1.14	1.19
		Hispanic	2,670	0.33	0.84	0.80	0.89	0.82	0.77	0.87	0.88	0.83	0.93
		Asian	1,420	0.22	0.53	0.50	0.57	0.60	0.56	0.64	0.74	0.69	0.79
	Competence	White	24,781	0.33	1.00	*Reference*		1.00	*Reference*		1.00	*Reference*	
		Black	23,213	0.56	1.46	1.43	1.49	1.66	1.62	1.70	1.50	1.47	1.54
		Hispanic	2,674	0.33	0.84	0.80	0.89	1.14	1.07	1.21	1.20	1.13	1.27
		Asian	1,575	0.24	0.73	0.69	0.78	0.86	0.81	0.91	0.95	0.89	1.01
Supporting Credibility	Competence	White	20,180	0.27	1.00	*Reference*		1.00	*Reference*		1.00	*Reference*	
		Black	8,236	0.2	0.72	0.69	0.74	0.85	0.82	0.88	0.82	0.79	0.85
		Hispanic	3,377	0.41	0.89	0.84	0.94	1.07	1.00	1.15	0.95	0.89	1.02
		Asian	1,913	0.3	1.27	1.20	1.34	1.37	1.30	1.45	1.30	1.23	1.38

^1^ Language supporting patient credibility were phrases endorsing competence such as “*good historian*” or “*reliable historian*”; Language undermining credibility fell into 2 categories: that which suggested insincerity (e.g., “patient *insists* the pain is still a 10” or “he is *adamant* that he took the medication”) and that which suggested incompetence (e.g., “*poor historian*” or “*inconsistent historian*”)

^2^ All models use GEE to account for clustering of notes within patient-clinician dyads

^3^ Model 1 adjusts for patient age, gender, insurance (private, Medicare, Medicaid, or uninsured), and use of interpreter

^4^ Model 2 adjusts for patient age, gender, insurance (private, Medicare, Medicaid, or uninsured), use of interpreter, diagnoses of substance use disorder and severe mental illness, type of clinician author (physician-in-training, attending physician, or advanced practice provider), and encounter department (emergency medicine, internal medicine, obstetrics/gynecology, or surgery)

In fully adjusted models, there was no difference between Hispanic/Latino vs. White patients in language undermining patient credibility overall (aOR 0.99, 95%CI 0.95–1.03). When examining terms related to sincerity and competence separately, our findings were bidirectional. Notes written about Hispanic/Latino vs. White patients had lower odds of undermining sincerity (aOR 0.88, 95%CI 0.83–0.93). In unadjusted analyses, notes written about Hispanic/Latino vs. White patients also had lower odds of undermining competence, but this relationship was reversed in the fully adjusted model (aOR 1.20; 95% CI 1.13–1.27).

Notes written about Asian vs. White patients had lower odds of having language undermining credibility overall (aOR 0.85, 95% CI 0.81–0.89). When examining terms related to sincerity and competence separately, in the fully adjusted models notes written about Asian vs. White patients had lower odds of language undermining sincerity (aOR 0.74, 95% CI 0.69–0.79) but did not differ in the prevalence of language undermining competence (aOR 0.95, 95% CI 0.89–1.01).

### Language supporting patient credibility

Language supporting patients’ credibility was found in 33,706 notes (0.26%). Notes written about Black compared to White patients had lower odds of language supporting credibility (aOR 0.82; 95% CI 0.79–0.85), whereas notes written about Asian vs. White patients had higher odds of supporting credibility (aOR 1.30, 95% CI 1.23–1.38). There was no difference in language supporting patient credibility in notes written about Hispanic/Latino vs. White patients.

## Discussion

We found evidence of racial bias in clinician assessments of patient credibility, based on the language they used in documenting clinical encounters. Specifically, after accounting for multiple potential confounders, we found that clinicians were more likely to insinuate in the EHR that Black compared to White patients were not truthful and not competent in reporting their own experiences. This testimonial injustice can harm Black individuals in numerous ways. Having one’s concerns dismissed has an immediate negative impact on the individual’s experience of respect, which erodes trust and decreases likelihood of engaging in future care or following treatment recommendations [12–14]. Further, failure to attend seriously to patients’ own reported symptoms may lead to physical harms such as untreated pain, delayed diagnoses, incorrect treatments, serious medical errors, and even death [21–25]. Because notes with biased language can affect the attitudes and decision-making of subsequent clinicians reading those notes [8], language undermining Black patients’ credibility may contribute to healthcare inequities.

We might question, in light of the low prevalence of credibility language detected by our models, whether the racial differences we observed, even if statistically significant as a result of our large sample size, are clinically important. We believe the language detected by our NLP models, which relied upon a limited set of specific words that explicitly cast doubt on the patient’s testimony, represents the tip of an iceberg in terms of how words are used to undermine patients’ credibility, and that our findings accordingly signal larger underlying disparities in credibility assessments. In our own qualitative work examining medical records [1], we have found doubt conveyed by clinicians in the medical record in more nuanced and implicit ways, such as placing the patient’s words in quotes (e.g., he states his pain is “still a 10”), juxtaposing the patient’s subjective report with their own objective observation (e.g., reports adherence to meds but doesn’t know Lasix dosing), or using a greater number of “evidential” terms that treat patient-reported information as “hearsay,” allowing the clinician to be noncommittal about believing that information (e.g., she says she has a headache vs. she has a headache). Our own and another prior study found higher use of quotes [14] and evidentials in the notes of Black compared to White patients [14,15], adding to evidence of a credibility deficit experienced by Black patients [26].

It has been suggested that the term ‘poor historian’ (or any of its variants) may be used by clinicians who themselves failed to obtain a good history [27–31]. Thus, disproportionate use of this term in the notes of Black patients may reflect, in addition to credibility bias, ineffectiveness in communicating with Black patients [12,13,32,33]. Importantly, use of the term ‘poor historian’ has the potential to bias future clinicians, who may discount the patient’s symptoms or be less likely to solicit the patient’s story. There are times when a clinician may reasonably be concerned that they weren’t able to gather the information they needed because the patient was not able to communicate it clearly. In those instances, we suggest specifying that to be the case – “patient unable to provide a complete history” or “patient is uncertain of some details” – that would convey less blame or avoid a global discrediting label.

One could argue that it may also be dangerous to not question the accuracy of a patient’s history when that patient is not being truthful, is misremembering, or has a recognized mental health disorder such as malingering. In these cases, it is not our intent to prohibit or criticize the conveying of such doubt in a medical record. We cannot and do not intend to assert that any individual clinician is biased against any individual patient simply because they use one of these words. Our primary concern here is that the observed racial differences in the use of these words suggest an implicit racial bias, on a population-level, in assessments of credibility. It is this bias to which we encourage clinicians to become attentive, both in terms of their own assessments of patient credibility as well as in interpretation of what has been written in the record by other clinicians. When examining specific credibility-related terms, we found that, after adjusting for demographic and clinical characteristics including substance use disorder and mental illness, all terms were used more often among Black vs non-Black patients, except for those related to narcotic or drug seeking. While we cannot discern from our data the reasons underlying this finding, we suspect clinicians may avoid using those terms for Black patients out of awareness of the racial stereotypes they perpetuate. If so, raising clinicians’ awareness of the biases and stereotypes that likely underlie the disproportionate use of other terms undermining Black patients’ credibility may help to remedy racial inequities in credibility assessments and credibility-related documentation.

Although we found consistent evidence of credibility bias against Black compared to White patients, we did not find similar evidence for Hispanic/Latino or Asian patients. Indeed, we found the opposite, with fewer terms questioning sincerity among Hispanic/Latino or Asian patients compared to Whites. While our data do not provide clear explanations for these findings, it may be that the sociocultural distance between clinicians and patients perceived as “foreign” [34] result in less in-depth, more superficial interactions and therefore fewer opportunities to suspect patients of insincerity. Similarly, Asian patients’ being more likely than White patients to be explicitly called out as “good historians” may reflect a lower expectation for accurate history provision among patients perceived as foreign. This represents a form of “reverse linguistic stereotyping,” in which a person’s race, ethnicity, or appearance influences expectations and perceptions of that person’s communication and may lead a clinician to praise articulate histories in the face of anticipated language or cultural barriers.[35] “Poor historian” terminology had a lower prevalence in unadjusted analyses for Hispanic/Latino patients; however this association was inverted after adjustment for demographics (primarily patient age), which may reflect greater language barriers among older Hispanic patients.[36] Further studies exploring clinician perceptions of patient credibility by race and ethnicity could test these hypothetical explanations. Based on our findings, it appears that credibility bias is worst for Black Americans.

Our study has several limitations. We studied records from one health system, although the system does expand across the mid-Atlantic region and includes a broad array of clinical settings (5 different hospitals, both community-based and academic, as well as a network of ambulatory practices) and disciplines. Our health system includes a higher proportion of Black patients than the national average, and we do not know whether disparities might be larger or smaller in other settings. We did not have data on clinicians beyond their role/training and clinical department and could not examine the influence of other clinician characteristics (e.g., age, race, gender). We also did not analyze nursing notes because, in our health system, they are mostly templated. These are important areas for future study. We selected a limited set of terms representing credibility language; as noted above, our findings may therefore underestimate its true prevalence [14]. We used NLP models with high, but not perfect, accuracy in detecting credibility-related language [16]; these models may have therefore misclassified some notes and thereby under- or overestimated the prevalence of credibility-related language. Any resulting misclassification, however, was likely to be non-differential; i.e., it is unlikely that the NLP models were less accurate in classifying credibility-related terms for one racial/ethnic group vs another. The impact of such misclassification, therefore, would be to bias toward more conservative (less pronounced) estimates of racial disparities. In addition, we did not capture evidence for supporting credibility in the ‘sincerity’ category because, while clinicians do often document that a patient is a good historian, there is no parallel documentation practice for complimenting the patient on being truthful. Finally, we had a relatively small proportion of notes from patients who were not identified as Black or White, and documentation of demographic data in the medical record is not always accurate.

In conclusion, clinician documentation undermining patient credibility may disproportionately stigmatize Black individuals and favor Asian individuals. As stigmatizing language in medical records has been shown to negatively influence clinician attitudes and decision-making [8,9], these racial differences in documentation may negatively influence quality of care and outcomes and further exacerbate health inequities. This specific type of bias related to credibility assessment has been relatively overlooked and has therefore remained unchecked. Medical educators have taken up efforts to train future physicians to engage in more respectful, patient-centered documentation, [37,38] and to learn about the structural inequities and interpersonal attitudes [39] that underlie biased language use in the EHR. We recommend that these curricula include content on testimonial injustice and suggest eliminating phrases such as ‘poor historian,’ as well as veiled insinuations about patient insincerity. As artificial intelligence applications are increasingly adopted to aid in note writing, consideration should be given to programming those applications to also avoid such language. Finally, we recommend that health professionals continue to confront the racial biases underlying differential assessments of patient credibility in the first place.

## Supporting information

S1 Appendix ICD-10 Codes for Substance Use Disorders and Severe Mental Illness. (DOCX)
