# Supplementary material for: Racial bias in clinician assessment of patient credibility: Evidence from electronic health records
Source: PLoS One. 2025 Aug 13;20(8):e0328134. doi: 10.1371/journal.pone.0328134 (PMC12349006; doi:10.1371/journal.pone.0328134)
Supplement: S1 Appendix — (DOCX) [file pone.0328134.s001.docx]

**Appendix. ICD-10 Codes for Substance Use Disorders and Severe Mental Illness.**

| **Condition** | **ICD-10 Codes** |
| --- | --- |
| Substance Use Disorders | F10, Z71.4, G31.2, G62.1, G72.1, I42.6, K29.2, K70, K86.0, O35.4, P04.3, Q86.0, F11-F16, F19, T40, T42.4, T43.6, Z71.5, P04.4, P96.1 |
| Severe Mental Illness | F20.x, F21.x, F22.x, F25.x, F28.x, F29.x, F30.x-F31.x, F32.2-F32.3, F33.2-F33.3 |

Sources:

Mansour H, Mueller C, Davis KAS, Burton A, Shetty H, Hotopf M, Osborn D, Stewart R, Sommerlad A. Severe mental illness diagnosis in English general hospitals 2006-2017: A registry linkage study. PLoS Med. 2020 Sep 17;17(9):e1003306. PMID: 32941435

Wang L, Homayra F, Pearce LA, Panagiotoglou D, McKendry R, Barrios R, Mitton C, Nosyk B. Identifying mental health and substance use disorders using emergency department and hospital records: a population-based retrospective cohort study of diagnostic concordance and disease attribution. BMJ Open. 2019 Jul 11;9(7):e030530. PMID: 31300509

Warren N, Leske S, Arnautovska U, Northwood K, Kisely S, Siskind D. Prevalence of frailty in severe mental illness: findings from the UK Biobank. BJPsych Open. 2023 Oct 12;9(6):e185. PMID: 37821357
